# Adipose-derived stromal cells reverse insulin resistance through inhibition of M1 expression in a type 2 diabetes mellitus mouse model

**DOI:** 10.1186/s13287-022-03046-0

**Published:** 2022-07-26

**Authors:** Lee-Wei Chen, Pei-Hsuan Chen, Chia-Hua Tang, Jui-Hung Yen

**Affiliations:** 1 Department of Surgery, Kaohsiung Veterans General Hospital, No.386, Ta-Chung 1st Road, Kaohsiung, 813 Taiwan; 2 Institute of Emergency and Critical Care Medicine, National Yang Ming Chiao Tung University, No.155, Sec.2, Linong Street, Taipei, 112 Taiwan; 3 Department of Biological Sciences, National Sun Yat-Sen University, No.70, Lien-Hai Road, Kaohsiung, 804 Taiwan; 4 Department of Microbiology and Immunology, Indiana University School of Medicine, 2101 E. Coliseum Blvd. Fort Wayne, Indianapolis, IN 46805 USA

**Keywords:** Stromal vascular fractions, Adipose tissue macrophages, IL-10, DPP4, Foxp3

## Abstract

**Background:**

Adipose tissue inflammation is considered as one of the major mechanisms underlying the pathogenesis of insulin resistance and complications in diabetes. Here, we aimed to study the effects of adipose-derived stromal cells on diabetes-induced insulin resistance and M1 cytokine expression.

**Methods:**

Stromal vascular fractions (SVFs) purified from the inguinal adipose tissue of diabetic mice were treated with plasma from either nondiabetic (*Lepr*^+*/*+^) or diabetic (*Lepr*^*db/db*^) mice and injected into the inguinal white adipose tissue of *Lepr*^*db/db*^ mice.

**Results:**

We found that diabetic plasma treatment induced, whereas nondiabetic plasma suppressed TNF-*α*, IL-1*β*, and dipeptidyl peptidase 4 (DPP4) mRNA expression in SVFs in vitro. Importantly, the injection of nondiabetic plasma-treated SVFs significantly decreased TNF-*α*, IL-6, IL-1*β*, CCL2, and IL-33 and induced IL-10 mRNA expression in adipose tissue of *Lepr*^*db/db*^ mice in vivo. Furthermore, we observed that nondiabetic plasma-treated SVFs increased mRNA expression of Foxp3 in adipose tissue macrophages and Foxp3 in adipose CD4^+^ T cells, decreased CD11b^+^CD11c^+^ cells in adipose tissue, and suppressed mRNA expression of ICAM-1, FCM3, IL-6, IL-1*β*, iNOS, TNF-*α*, and DPP4 as well as protein expression of DPP4 and phosphorylated JNK and NF-*κ*B in the liver of *Lepr*^*db/db*^ mice. Moreover, we found that nondiabetic plasma-treated SVFs increased Akt activation following insulin administration and attenuated glucose intolerance in *Lepr*^*db/db*^ mice.

**Conclusions:**

Our results demonstrate that nondiabetic plasma inhibits M1 but increases M2 cytokine expression in adipose tissue of diabetic mice. Most importantly, our findings reveal that nondiabetic plasma-treated SVFs are capable of mitigating diabetes-induced plasma DPP4 activity, liver inflammation, and insulin resistance and that may be mediated through suppressing M1 cytokines but increasing IL-10 and Tregs in adipose tissue. Altogether, our findings suggest that adipose stromal cell-based therapy could potentially be developed as an efficient therapeutic strategy for the treatment of diabetes.

**Supplementary Information:**

The online version contains supplementary material available at 10.1186/s13287-022-03046-0.

## Background

Chronic inflammation induced by immune cells such as macrophages is an important factor triggering the dysregulation of metabolic homeostasis [1]. A close correlation has been demonstrated between adipose tissue inflammation and metabolic diseases in obesity [2, 3]. Studies have shown that the dysregulation of M1/M2 polarization in adipose tissue represents one of the major mechanisms underlying the pathogenesis of obesity and comorbidities, such as insulin resistance and nonalcoholic fatty liver disease [1]. In the case of obesity, adipose tissue macrophages (ATMs) alter their phenotype from anti-inflammatory M2 to the proinflammatory M1 ATMs. These M1 macrophages produce inflammatory cytokines, such as TNF-α, IL-6, and IL-1β, to activate inflammatory pathways in insulin target cells that subsequently activate JNK and NF-κB pathways [4]. Notably, the depletion of M1 macrophages increases the sensitivity to insulin in obese mice, whereas the reduction of M2 macrophages predisposes to insulin resistance in lean mice [2]. Furthermore, chronic inflammation results in vascular and kidney complications in patients with diabetes [5]. Although the inflammatory responses can be stimulated by various mechanisms, including hyperglycemia-induced cell death that increases the aggregation of macrophages in the kidney [6], the mechanisms of M1 polarization in adipose tissue under chronic inflammation and related complications in diabetes have not been well characterized.

The role of M2 cytokine IL-10 in modulating metabolic dysfunction has been demonstrated. Studies have shown that endogenous IL-10 is a protective factor against diet-induced insulin resistance in the liver [7]. Furthermore, the inhibition of IL-10 leads to increased expression of inflammatory cytokines, worsened insulin signaling, and activated glucogenic and lipogenic pathways [7]. Moreover, ATM-derived IL-10, induced by insulin and lipopolysaccharides, has been shown to suppress hepatic glucose production in cooperation with insulin [8]. However, the therapeutic values of IL-10 for the treatment of diabetes-related complications need to be clarified and require further investigation.

Obesity is associated with chronic low-grade inflammation in adipose tissue, and the increased number of ATMs is strongly linked to the onset of type 2 diabetes. A relative high number of regulatory T cells (Tregs) (40% of T cells) are present in adipose tissue compared to other lymphoid tissues in mice [9]. Tregs, characterized by the expression of transcription factor, forkhead box P3 (Foxp3), provide the critical defense against abnormal immune responses, such as allergy, inflammation, and infection [9]. Tregs can also suppress the activation and proliferation of effector T cells and regulate the activity of the innate immune system. The study has found that the number of Tregs in epididymal fat is markedly reduced in obese animals, and this reduction is closely associated with the development of insulin resistance [10]. However, currently the regulatory mechanisms of Tregs in adipose tissue inflammation of diabetes have not been fully elucidated.

In the current study, we examined the effect of nondiabetic plasma- and diabetic plasma-treated diabetic adipose-derived stromal vascular fractions (SVFs) on adipose tissue inflammation, systemic inflammation, liver inflammation, and insulin resistance in diabetic mice. We hypothesized that nondiabetic plasma-treated diabetic SVFs can potentially attenuate diabetes-induced inflammatory mediators, systemic inflammation, and insulin resistance through downregulating inflammatory M1 but upregulating anti-inflammatory M2 cytokine expression in adipose tissue. Thus, we examined whether the injection of nondiabetic plasma-treated diabetic SVFs modulated plasma cytokine level and activity, liver proinflammatory cytokine expression, and glucose intolerance in diabetic mice.

## Methods

### Mice

*Lepr*^*db/*+^ mice were purchased from the Jackson Laboratory (Bar Harbor, ME) and bred to obtain diabetic *Lepr*^*db/db*^ and nondiabetic *Lepr*^+/+^ mice. *Lepr*^*db/db*^ mice with a mutation in the gene encoding the leptin receptor become obese at 3 to 4 weeks of age and exhibit elevated plasma insulin and blood sugar at 4 to 8 weeks of age. *Lepr*^+/+^ and *Lepr*^*db/db*^ mice were fed with a standard laboratory diet (1324 TPF; Atromin; Large Germany; 11.9 kJ/g, 19% crude protein, 4% crude fat, 6% crude fiber) and had ad libitum access to water and food. All animal experimental procedures were designed, performed, and approved by the Institutional Animal Care and Use Committee (IACUC) at Kaohsiung Veterans General Hospital.

### Preparation of stromal vascular fractions (SVFs)

Vascular adipose tissue isolated from bilateral inguinal adipose tissue of *Lepr*^*db/db*^ and *Lepr*^+*/*+^ mice at 10 to 12 weeks of age was minced into small pieces and then digested with collagenase 8 (Sigma-Aldrich, Cat# C2139) in ice-cold HBSS (2 mg/ml) for 15 min at 37 °C. Cells were then passed through 100-μm cell strainers and centrifuged at 1,200 rpm for 10 min. The cell pellets were then collected and retrieved as SVFs for experiments. The number of SVF cells was counted with Cellometer (Nexcelom Bioscience). We harvested 3.4 ± 0.8 g inguinal adipose tissue from each *Lepr*^*db/db*^ mouse. Our system yields 1 × 10^7^ cells per gram of adipose tissue.

### In vitro treatment of SVFs and in vivo treatment of SVFs in Lepr^db/db^ mice

For in vitro treatment, plasma was prepared by dilution with HBSS (Hanks' balanced saline solution, Sigma-Aldrich, Cat# H6648) containing 0.5% BSA and 2 mM EDTA to achieve a 10% final concentration. Prepared *Lepr*^*db/db*^ or *Lepr*^+*/*+^ plasma was added to collected *Lepr*^*db/db*^ SVFs to make the final concentration of 2 × 10^7^ cells/ml. The samples were then incubated at 37 °C for 3.5 h as previously suggested [11]. After incubation, the samples were then centrifuged at 2,500 rpm for 5 min and washed with phosphate-buffered saline (PBS). Following centrifugation, the pellets were harvested and subjected to analysis. For in vivo studies***, Lepr***^*db/db*^ mice were randomly divided into three groups. Group I received PBS injection into the adipose tissue of the bilateral inguinal area as controls. Group II and group III received the injection of *Lepr*^*db/db*^ plasma- or *Lepr*^+*/*+^ plasma-treated *Lepr*^*db/db*^ SVFs (2 × 10^7^ cells), respectively, into the adipose tissue of the bilateral inguinal area. At day 7 post-injection, the animals were killed, and the liver, adipose tissue, and plasma were then harvested for analysis.

### Isolation of ATMs from SVFs with microbeads

The SVFs were pelleted by centrifugation, washed once with buffer, and incubated with anti-F4/80 microbeads (130–110-443, Miltenyi Biotec). The cells were then rinsed once with PBS–bovine serum albumin (BSA), pelleted by centrifugation, and resuspended in PBS–BSA. ATMs were isolated using magnetic separation columns, and non-ATMs (NATMs) were collected from the washing solution.

### RNA isolation and quantitative real-time polymerase chain reaction (Q-PCR)

Total RNA was purified from samples using total RNA Miniprep Purification Kits (GeneMark) and then reverse-transcribed into cDNA using RT kits (Invitrogen, Carlsbad, CA, Lot# 2,234,812). For Q-PCR assay, 2 µl of 200 ng cDNA was added into a mixture containing 12.5 μl of 2 × Fast SYBR Green Master Mix (Applied biosystems, Cat# 4,385,612), 2.5 μl of sense and anti-sense primers (25 μM), and 8 μl of sterile water to reach the final volume of 25 µl. The amplification was performed by using StepOnePlus™ Real-Time PCR System (Applied Biosystems 7300).

### Western immunoblots

The expression of pAkt (Cell Signaling, # 4060), Akt (Cell Signaling, # 4691), NF-κB (Cell Signaling, # 8242), pNF-κB p65 (Cell signaling, # 3033), JNK (Cell signaling, # 9252), pJNK (Cell signaling, # 9251), ERK (Cell Signaling, # 4695), pERK (Cell Signaling, # 9101), and DPP4 (GeneTex, # GTX84602) in the liver was detected by western immunoblot analysis. Briefly, the harvested tissues were homogenized in protein extraction buffer (Sigma), containing proteinase inhibitor cocktail (Roche), and then subjected to SDS-PAGE at 50 to 100 V for 2 h. Following transferring onto the nitrocellulose membrane, the membrane was blocked with 5% nonfat milk in TBST buffer (10 mM Tris–HCl, pH 7.5, 150 mM NaCl, and 1.2% Tween 20) for 1 h and incubated with the specific primary antibody at room temperature for 1 h. After washing with TBST buffer, the membrane was incubated with the secondary antibody. Following wash, the protein bands were identified by enhanced chemiluminescence (ECL) detection reagent (Millipore).

### Flow cytometry analysis

Cells in the SVFs were suspended in the staining buffer (PBS containing 0.5% BSA and 2 Mm ethylenediaminetetraacetic acid) and then incubated with CD11b (BioLegend, clone: M1/70) and CD11c (BioLegend, clone: N418) antibodies or the control isotypes at 4 °C. Thirty minutes later, cells were then washed twice and resuspended in the staining buffer. After incubation with 7-amino-actinomycin D (BioLegend), the cells were analyzed by Attune NxT Flow Cytometer (ThermoFisher). The data analysis was performed by using FlowJo (Tree Star, Ashland, OR). Cells with double positive expression of CD11b and CD11c were identified as M1 macrophages. For intracellular FoxP3 staining, PE/Cyanine7 anti-mouse CD4 (BioLegend, # 100,528)-stained cells were resuspended in 1 ml of the True-Nuclear™ 1X Fix Concentrate to each tube, vortex and incubate at room temperature in the dark for 60 min, followed by adding 2 mL of the True-Nuclear™ 1X Perm Buffer (BioLegend, # 424,401) to each tube and then centrifuge tubes at 2100 rpm at room temperature for 8 min, and discard the supernatant. Tubes were decanted, blotted, washed twice with True-Nuclear™ 1X Perm Buffer. Resuspend the cell pellet in 100 µl of the True-Nuclear™ 1X Perm Buffer. For detection of intracellular antigen, add 1 μl of PE–anti-mouse FoxP3 antibody (BioLegend, # 126,404) to each tube and incubate in the dark at room temperature for 60 min.

### Insulin treatment

For insulin injection, we fasted the mice for 16 h, injected the mice with either phosphate-buffered saline or insulin at 1.25 mIU/g body weight, and waited for 20 min before killing the mice and harvesting the livers.

### Plasma DPP4 activity

Plasma was harvested from cardiac blood and stored at − 20 °C until assayed. The activity of plasma DPP4 was measured by DPP4 assay kit (BioVision, Milpitas, CA, # K779-100). DPP4 cleaves substrates that results in releasing the quenched fluorescent group, AMC (7-amino-4-methyl coumarin), which can then be detected at Ex/Em = 360/460 nm by using a fluorescence reader.

#### Enzyme‑linked immunosorbent assay (ELISA)

CCL2, IL-33, and adiponectin were detected by using mouse ELIS kits from LSBio (# LS-F388), R&D systems (# DY479-05), and Invitrogen (# KMP0041), respectively. Briefly, the blood was harvested and centrifuged at 1000 × *g*, 4 °C for 15 min followed by serum collection. The ELISA plates were coated with 100 μl capture antibody at 4 °C overnight. Following wash and blocking, the samples and serial dilutions of standards were added into the plates and incubated at 4 °C for overnight. The samples were then incubated with the detection antibody followed by avidin-HRP incubation at room temperature for 30 min. The substrate, 3,3′,5,5′-tetramethylbenzidine (TMB), was then added and incubated for 15 min followed by adding stop solution to stop reaction. The plate was then subjected to measurement of absorbance at 450 nm by using an ELISA reader.

#### Intraperitoneal glucose tolerance tests (IPGTTs)

IPGTTs were conducted to test glucose tolerance. Mice were subjected to a period of 15 h fasting, and blood glucose was then measured before and at 15, 30, 45, 60, 75, 90, and 120 min after intraperitoneal injection of glucose (1 g/kg body weight; Sigma). Plasma glucose was measured by using a glucose meter (Accu-Chek Performa; Roche, Switzerland).

#### Statistical analysis

Data were analyzed by unpaired *t* test for the comparisons between two groups or by one-way analysis of variance (ANOVA) followed by Tukey’s multiple comparison test for the comparisons between multiple groups. Data are presented as mean ± standard error. Statistical significance was determined as *p* < 0.05.

## Results

### Diabetic plasma increases M1 cytokine expression, but nondiabetic plasma reduces M1 and increases M2 cytokine expression in SVFs of ***Lepr***^***db/db***^ mice in vitro

**Fig. 1:**
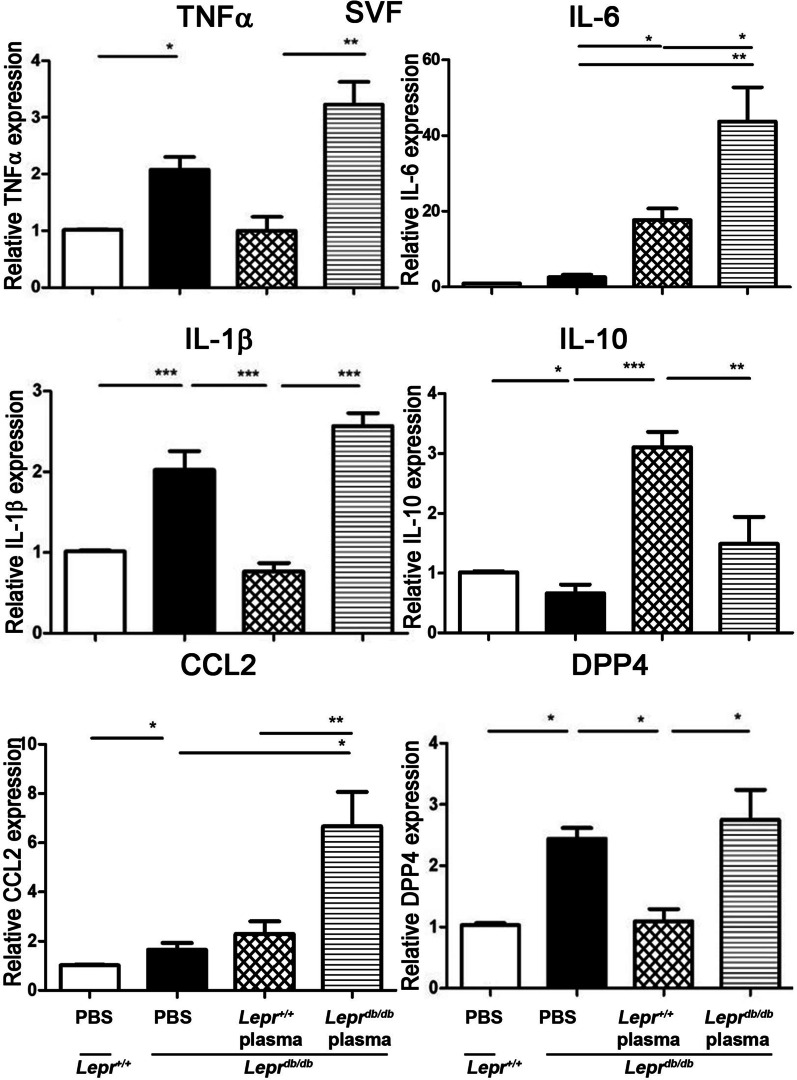
Diabetic plasma increases M1 cytokine expression, but nondiabetic plasma reduces M1 and increases M2 cytokine expression in *Lepr*^*db/db*^ SVFs. SVFs were harvested from the adipose tissue of *Lepr*^+*/*+^ mice and treated with PBS or from the adipose tissue of *Lepr*^*db/db*^ mice and treated with PBS or plasma from *Lepr*^*db/db*^ or *Lepr*.^+*/*+^ mice in vitro. 3.5 h hours after treatment, cells were then collected and subjected to Q-PCR analysis for mRNA expression of TNFα, IL-6, IL-1β, IL-10, CCL2, and DPP4. *N* = 5/group. *p < 0.05, **p < *0.01*, ***p < 0.001

M1 ATMs produce proinflammatory cytokines, such as TNF-α, IL-6, and CCL2. On the other hand, M2 ATMs are characterized by the relatively high expression of IL-10 [12]. To examine the effects of diabetic and nondiabetic plasma on M1 and M2 cytokine expression in SVFs of the inguinal adipose tissue from type 2 diabetic (*Lepr*^*db/db*^) mice, SVFs harvested from the adipose tissue of *Lepr*^*db/db*^ mice were treated with PBS or plasma from *Lepr*^*db/db*^ or *Lepr*^+*/*+^ mice for 3.5 h followed by Q-PCR analysis of M1 and M2 cytokine expression. Previous study demonstrated that 10% (v/v) plasma from diet-induced obesity (DIO) mice induced higher expression of both CCL2 and IL-6 mRNA than plasma from lean mice in SVFs from DIO mice, but did not have this effect on SVFs from lean mice [11]. We harvested 3.4 ± 0.8 g inguinal adipose tissue from *Lepr*^*db/db*^ mice. We first compared the cytokine expression in SVFs from diabetic (*Lepr*^*db/db*^) and nondiabetic (*Lepr*^+*/*+^) mice. SVFs from *Lepr*^*db/db*^ mice exhibited a significant increase of TNF-α, IL-1β, CCL2, and DPP4, but a reduction of IL-10 mRNA expression compared to SVFs from *Lepr*^+*/*+^ mice (Fig. 1). Notably, we observed that treatment of *Lepr*^*db/db*^ plasma upregulated the expression of inflammatory molecules, including TNFα, IL-1β, CCL2, IL-6, and DPP4, compared to the treatment of PBS in *Lepr*^*db/db*^ SVFs. In contrast, *Lepr*^+*/*+^ plasma treatment downregulated TNFα, IL-1β, and DPP4, but upregulated IL-10 expression compared to PBS treatment in *Lepr*^*db/db*^ SVFs. Importantly, the treatment of *Lepr*^+*/*+^ plasma resulted in a significant downregulation of TNF-α, IL-1β, CCL2, IL-6, and DPP4, but an upregulation of IL-10 expression compared to the treatment of *Lepr*^*db/db*^ plasma in *Lepr*^*db/db*^ SVFs (Fig. 1). Collectively, these results suggest that nondiabetic SVFs exhibited an increased expression of M2 cytokine but a decreased expression of M1 cytokines compared to the diabetic SVFs. Most importantly, our results reveal that plasma harvested from nondiabetic mice is capable of suppressing M1 cytokines but enhancing M2 cytokine in diabetic SVFs.

### Nondiabetic plasma-treated SVFs suppress M1 but promote M2 cytokine expression in the adipose tissue of ***Lepr***^***db/db***^ mice in vivo

**Fig. 2:**
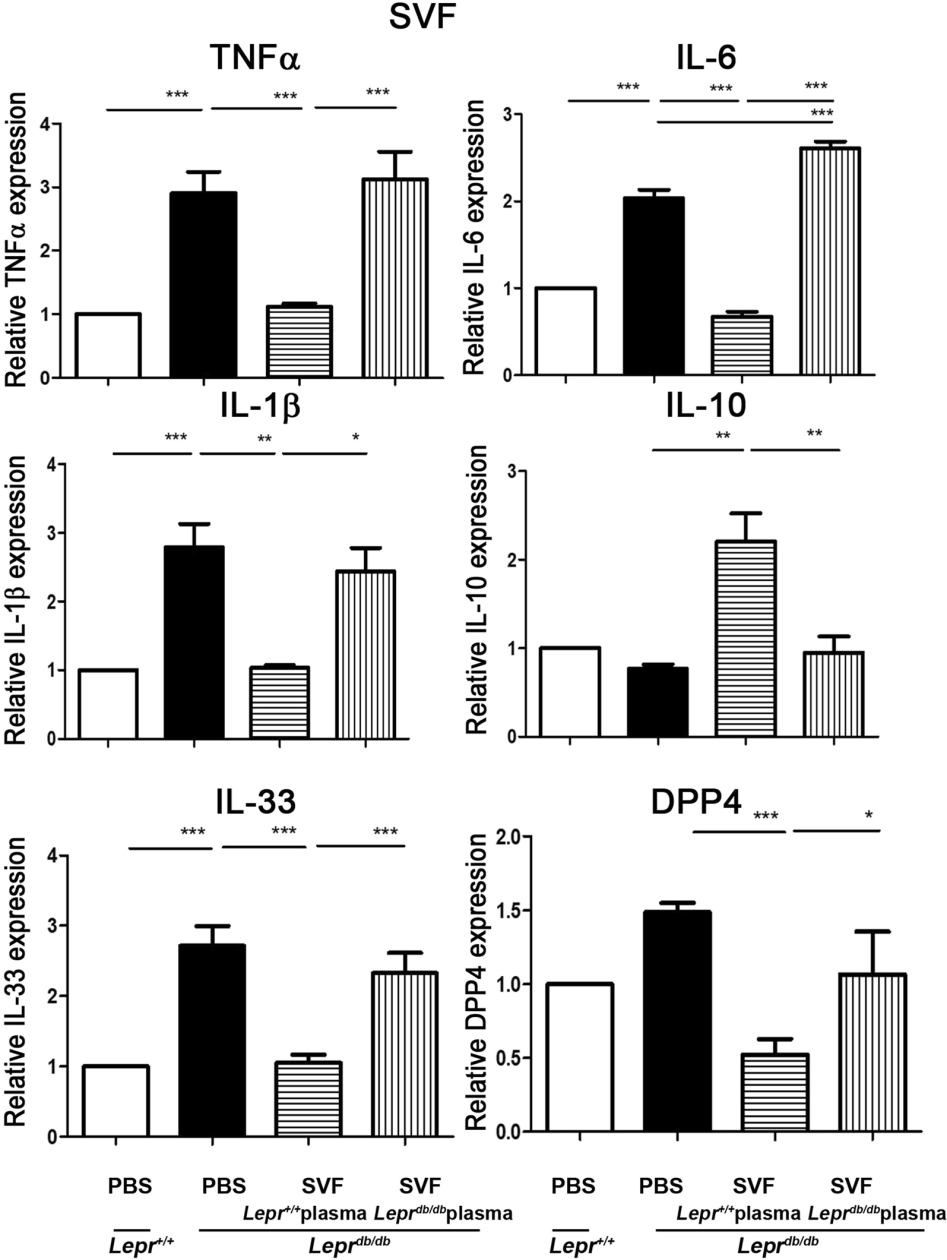
Nondiabetic plasma-treated SVFs suppress M1 but promote IL-10 mRNA expression in the adipose tissue of *Lepr*^*db/db*^ mice. SVFs were harvested from adipose tissue of *Lepr*^*db/db*^ mice and treated with plasma from *Lepr*^+*/*+^ or *Lepr*^*db/db*^ mice. *Lepr*^+*/*+^ plasma- or *Lepr*^*db/db*^ plasma-treated SVFs were injected into the inguinal WAT of *Lepr*^*db/db*^ mice. *Lepr*^+*/*+^ and *Lepr*^*db/db*^ mice were also treated with PBS as controls. One week later, the inguinal WAT was harvested from *Lepr*^+*/*+^ mice treated with PBS and *Lepr*^*db/db*^ mice treated with PBS, *Lepr*^+*/*+^ plasma-treated SVFs, or *Lepr*.^*db/db*^ plasma-treated SVFs. SVFs were then harvested from WAT followed by Q-PCR analysis for mRNA expression of TNFα, IL-1β, IL-33, IL-6, IL-10, and DPP4. *N* = 5/group. *p < 0.05, **p < *0.01*, ***p < *0.001*

Since we observed that nondiabetic plasma promoted M2 phenotype in diabetic SVFs (Fig. 1), we examined whether nondiabetic plasma-treated SVFs could be a source of cell therapy to reverse diabetes-induced M1 expression in adipose tissue of *Lepr*^*db/db*^ mice. SVFs isolated from the adipose tissue of *Lepr*^*db/db*^ mice were treated with plasma harvested from *Lepr*^+*/*+^ or *Lepr*^*db/db*^ mice and then injected into the inguinal white adipose tissue (WAT) of *Lepr*^*db/db*^ mice. In addition, *Lepr*^+*/*+^ and *Lepr*^*db/db*^ mice were treated with PBS as controls. One week later, the inguinal WATs of PBS- and SVFs-administered recipients were harvested, and SVFs were isolated from the inguinal WATs followed by the measurement of cytokine expression. Our results showed that SVFs harvested from the inguinal WAT of *Lepr*^*db/db*^ mice receiving PBS injection exhibited an increased expression of TNF-α, IL-1β, IL-33, IL-6, and DPP4 compared to those harvested from *Lepr*^+*/*+^ mice receiving PBS injection (Fig. 2). Importantly, we found that the injection of *Lepr*^+*/*+^ plasma-treated SVFs into the adipose tissue of *Lepr*^*db/db*^ mice significantly decreased the expression of M1 cytokines, TNFα, IL-1β, IL-33, IL-6, and DPP4, but increased the expression of M2 cytokine IL-10 in the SVFs of inguinal WATs compared to PBS-treated *Lepr*^*db/db*^ controls. However, the injection of *Lepr*^*db/db*^ plasma-treated SVFs into the adipose tissue of *Lepr*^*db/db*^ mice did not alter the expression of aforementioned M1 and M2 cytokines except slightly enhanced IL-6 expression compared to PBS-treated *Lepr*^*db/db*^ controls. Altogether, these results suggest that nondiabetic plasma-conditioned SVFs are capable of inhibiting M1 but promoting M2 cytokine expression in the adipose tissue of diabetic mice in vivo.

### Nondiabetic plasma-treated SVFs decrease CCL2 and DPP4 but increase IL-10 expression in ATMs of ***Lepr***^***db/db***^ mice

**Fig. 3:**
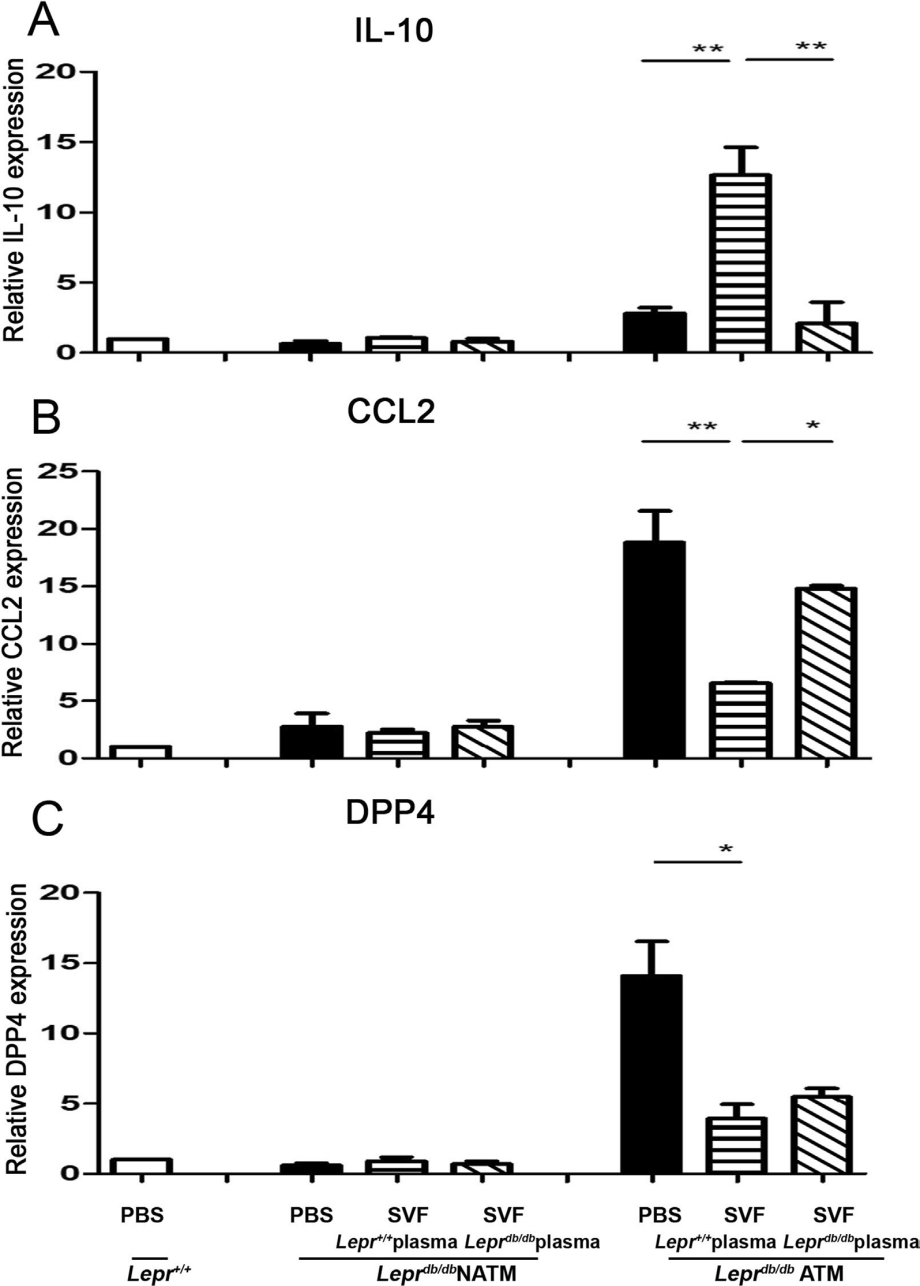
Nondiabetic plasma-treated SVFs decrease CCL2 and DPP4, and increase IL-10 mRNA expressions in the ATMs of *Lepr*^*db/db*^ mice. *Lepr*^*db/db*^ SVFs were treated with *Lepr*^+*/*+^ or *Lepr*^*db/db*^ plasma and then injected into the inguinal WAT of *Lepr*^*db/db*^ mice. *Lepr*^+*/*+^ and *Lepr*.^*db/db*^ mice were also treated with PBS as controls. One week later, ATMs and NATMs were isolated from the adipose tissue using microbeads and then subjected to Q-PCR analysis for IL-10 (**A**), CCL2 (**B**), and DPP4 (**C**) mRNA expression. *N* = 5/group. *p < 0.05, **p < 0.01

To examine whether nondiabetic plasma-treated SVFs affect M1 and M2 cytokine expression in ATMs in diabetic mice, *Lepr*^*db/db*^ SVFs treated with *Lepr*^+*/*+^ or *Lepr*^*db/db*^ plasma were injected into the adipose tissue of *Lepr*^*db/db*^ mice, and ATMs and NATMs were harvested from the adipose tissue followed by Q-PCR analysis to assess the expression of M1 and M2 cytokines. *Lepr*^+*/*+^ and *Lepr*^*db/db*^ mice treated with PBS were used as controls. Our results showed that the injection of *Lepr*^+*/*+^ plasma-treated SVFs into the adipose tissue of *Lepr*^*db/db*^ mice decreased CCL2 but increased IL-10 mRNA expression in the ATMs of *Lepr*^*db/db*^ mice compared to *Lepr*^*db/db*^ mice treated with PBS or *Lepr*^*db/db*^ plasma-treated *Lepr*^*db/db*^ SVFs (Fig. 3A and B). In addition, we observed that ATMs isolated from *Lepr*^*db/db*^ mice injected with *Lepr*^+*/*+^ plasma-treated SVFs or *Lepr*^*db/db*^ plasma-treated SVFs exhibited decreased DPP4 mRNA expression compared to those isolated from *Lepr*^*db/db*^ mice treated with PBS, although we did not observe a difference in DPP4 expression in ATMs isolated from *Lepr*^*db/db*^ mice injected with *Lepr*^+*/*+^ plasma-treated SVFs or *Lepr*^*db/db*^ plasma-treated SVFs (Fig. 3C). Finally, we found that *Lepr*^+*/*+^ or *Lepr*^*db/db*^ plasma-treated SVFs did not alter M1 and M2 expression in NATMs isolated from *Lepr*^*db/db*^ mice compared to those isolated from PBS-treated *Lepr*^*db/db*^ controls. Collectively, our results demonstrate that nondiabetic plasma-treated SVFs repress M1 but promote M2 phenotype of ATMs in *Lepr*^*db/db*^ mice.

### Nondiabetic plasma-treated SVFs enhance Foxp3 expression in ATMs of ***Lepr***^***db/db***^ mice

**Fig. 4:**
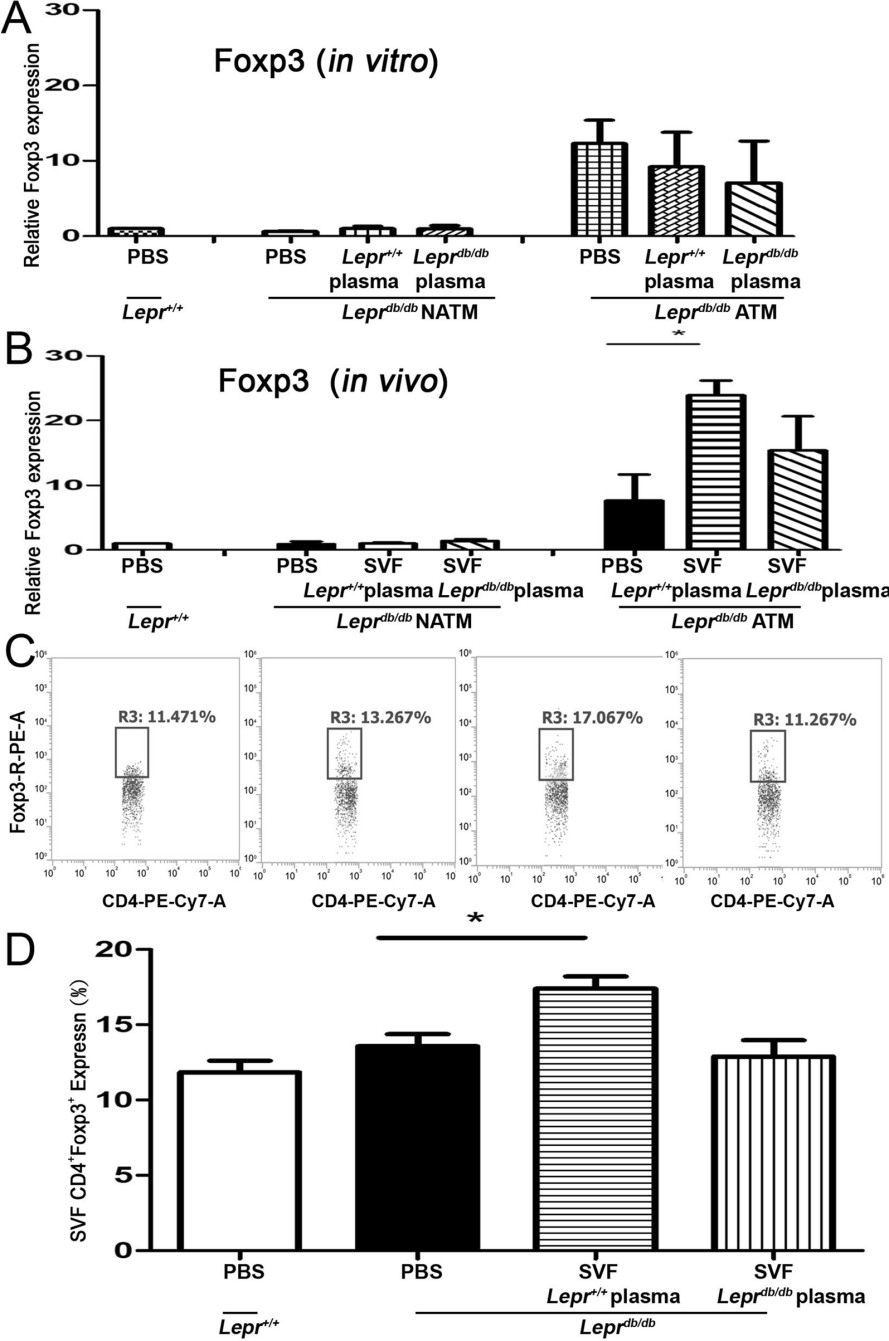
Nondiabetic plasma-treated SVFs increase Foxp3 expression and CD4^+^ Tregs in the adipose tissue of *Lepr*^*db/db*^ mice. (**A**) *Lepr*^*db/db*^ SVFs were treated with PBS, *Lepr*^+*/*+^ plasma, or *Lepr*^*db/db*^ plasma, and *Lepr*^+*/*+^ SVFs were treated with PBS. 3.5 h after treatment, ATMs and NATMs were isolated from SVFs using the microbeads followed by Q-PCR analysis for Foxp3 mRNA expression. (**B**) *Lepr*^*db/db*^ SVFs were treated with *Lepr*^+*/*+^ or *Lepr*^*db/db*^ plasma and then injected into the inguinal WAT of *Lepr*^*db/db*^ mice. *Lepr*^+*/*+^ and *Lepr*^*db/db*^ mice were also treated with PBS as controls. One week later, ATMs and NATMs were isolated from adipose tissue using the microbeads and then subjected to Q-PCR analysis for Foxp3 mRNA expression. (**C** and **D**) Flow cytometry analysis was used to assess the frequency and number of CD4.^+^ Tregs in the adipose tissue. *N* = 5/group. *p < 0.05

The induction of IL-10 has been shown to promote Treg differentiation [13]. We therefore decided to evaluate whether nondiabetic plasma-treated SVFs that upregulate IL-10 expression could promote Treg differentiation in the adipose tissue of diabetic mice. Tregs were characterized by their expression of Foxp3. We first determined whether plasma isolated from *Lepr*^+*/*+^ or *Lepr*^*db/db*^ mice has a direct effect on modulating Foxp3 expression in the ATMs isolated from *Lepr*^*db/db*^ mice in vitro. We found that the treatment of *Lepr*^+*/*+^ or *Lepr*^*db/db*^ plasma did not alter Foxp3 expression in ATMs isolated from *Lepr*^*db/db*^ mice compared to those isolated from *Lepr*^*db/db*^ mice treated with PBS (Fig. 4A). We then investigated whether *Lepr*^+*/*+^ or *Lepr*^*db/db*^ plasma-treated SVFs would have an effect on Treg differentiation in vivo. *Lepr*^+*/*+^ or *Lepr*^*db/db*^ plasma-treated SVFs were injected into the inguinal WAT of *Lepr*^*db/db*^ mice, and the expression of Foxp3 was determined in ATMs of *Lepr*^*db/db*^ mice. Remarkably, we observed that the expression of Foxp3 in ATMs was significantly increased in *Lepr*^*db/db*^ mice injected with *Lepr*^+*/*+^ plasma-treated SVFs compared to those injected with PBS (Fig. 4B). Although there was a trend of increased Foxp3 expression in ATMs of *Lepr*^*db/db*^ mice injected with *Lepr*^*db/db*^ plasma treated with SVFs compared to those injected with PBS, it did not reach statically significant difference (Fig. 4B). In addition, we observed the expression of Foxp3 was very low in NATMs from *Lepr*^*db/db*^ mice injected with PBS, *Lepr*^+*/*+^ plasma treated with SVFs, or *Lepr*^*db/db*^ plasma -treated SVFs. Altogether, these results suggest that nondiabetic plasma-treated SVFs are capable of enhancing Foxp3 expression in ATMs of *Lepr*^*db/db*^ mice in vivo.

### Nondiabetic plasma-treated SVFs increase CD4^+^ Tregs in adipose tissues of ***Lepr***^***db/db***^ mice

To further examination whether increased Foxp3 expression in *Lepr*^*db/db*^ mice injected with *Lepr*^+*/*+^ plasma-treated SVFs influenced CD4^+^ Tregs of adipose tissue, we conducted flow cytometry analysis to assess the number of CD4^+^ Foxp3^+^ cells in the adipose tissue. We found that the number of CD4^+^ Foxp3^+^ cells was significantly increased in *Lepr*^*db/db*^ mice injected with *Lepr*^+*/*+^ plasma-treated SVFs compared to those injected with PBS (Fig. 4C and D). Collectively, these results suggest that nondiabetic plasma-treated SVFs increase CD4^+^ Tregs in the adipose tissue of diabetic mice.

### Nondiabetic plasma or diabetic plasma-treated SVFs decrease M1 macrophages in adipose tissues of ***Lepr***^***db/db***^ mice

**Fig. 5:**
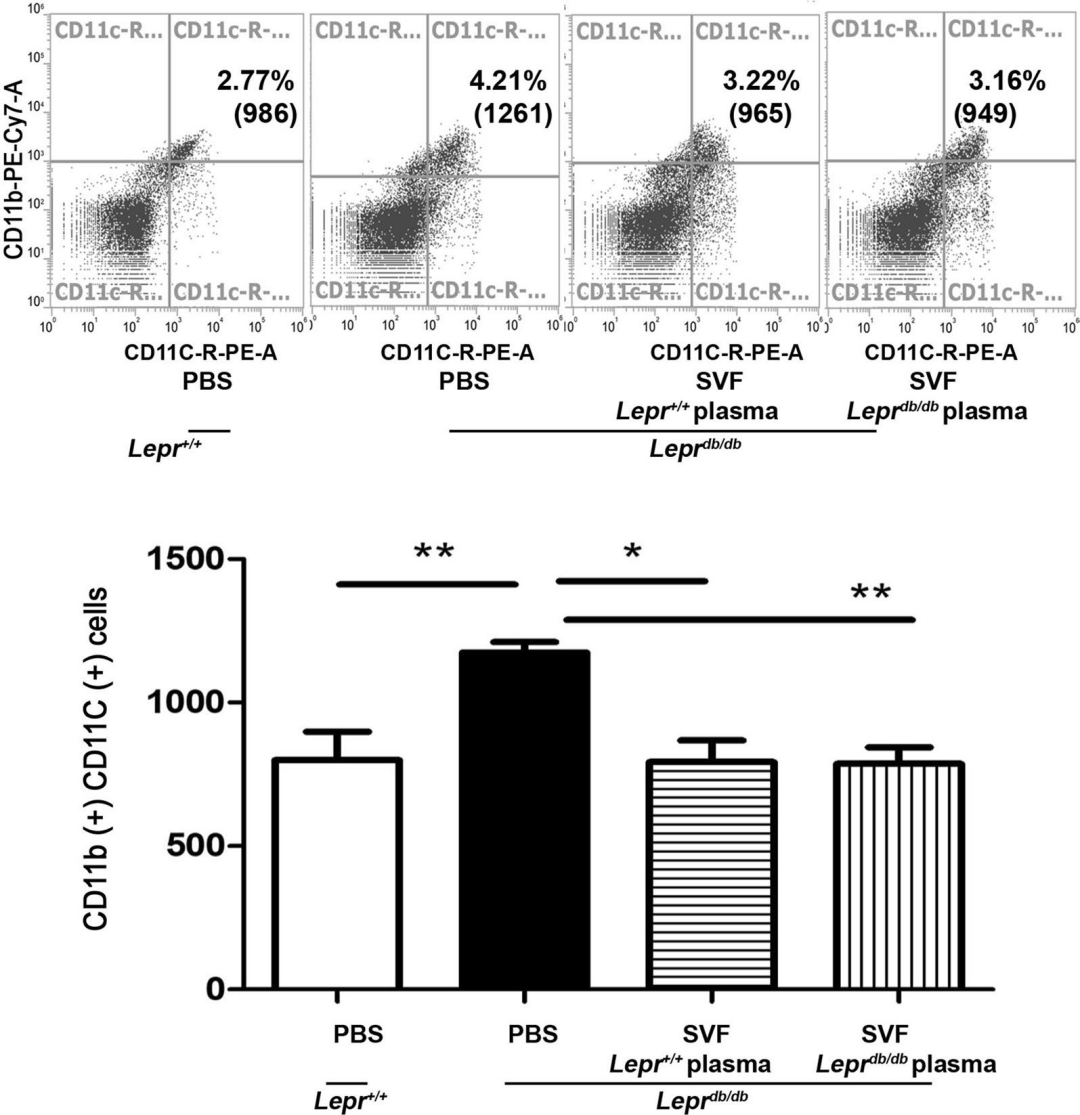
Nondiabetic plasma or diabetic plasma-treated SVFs decrease M1 macrophages in adipose tissues of *Lepr*^*db/db*^ mice. SVFs were harvested from the adipose tissue of *Lepr*^*db/db*^ mice and treated with plasma from *Lepr*^+*/*+^ or *Lepr*^*db/db*^ mice. *Lepr*^+*/*+^ or *Lepr*^*db/db*^ plasma-treated SVFs were injected into the inguinal WAT of *Lepr*^*db/db*^ mice. *Lepr*^+*/*+^ and *Lepr*^*db/db*^ mice were also treated with PBS as controls. One week later, we conducted flow cytometry analysis to assess the frequency and number of CD11c^+^ and CD11b.^+^ M1 macrophages in the adipose tissue. *N* = 5/group. *p < 0.05

To further examination whether increased Foxp3 expression in *Lepr*^*db/db*^ mice injected with *Lepr*^+*/*+^ plasma-treated SVFs influenced the differentiation of M1 macrophages, we conducted flow cytometry analysis to assess the frequency and number of CD11c^+^ and CD11b^+^ M1 macrophages in the adipose tissue. We found that the number of CD11c^+^ CD11b^+^ cells was significantly decreased in *Lepr*^*db/db*^ mice injected with *Lepr*^+*/*+^ plasma-treated SVFs compared to those injected with PBS. The number of CD11c^+^ CD11b^+^ cells was also significantly decreased in *Lepr*^*db/db*^ mice injected with *Lepr*^*db/db*^ plasma-treated SVFs compared to those injected with PBS (Fig. 5). Collectively, these results suggest that nondiabetic plasma-treated SVFs increase Treg cells and decrease M1 macrophages in the adipose tissue of diabetic mice.

### Nondiabetic plasma-treated SVFs decrease the expression of inflammatory meditators and suppress the activation of JNK and NFκB in the liver of ***Lepr***^***db/db***^ mice

**Fig. 6:**
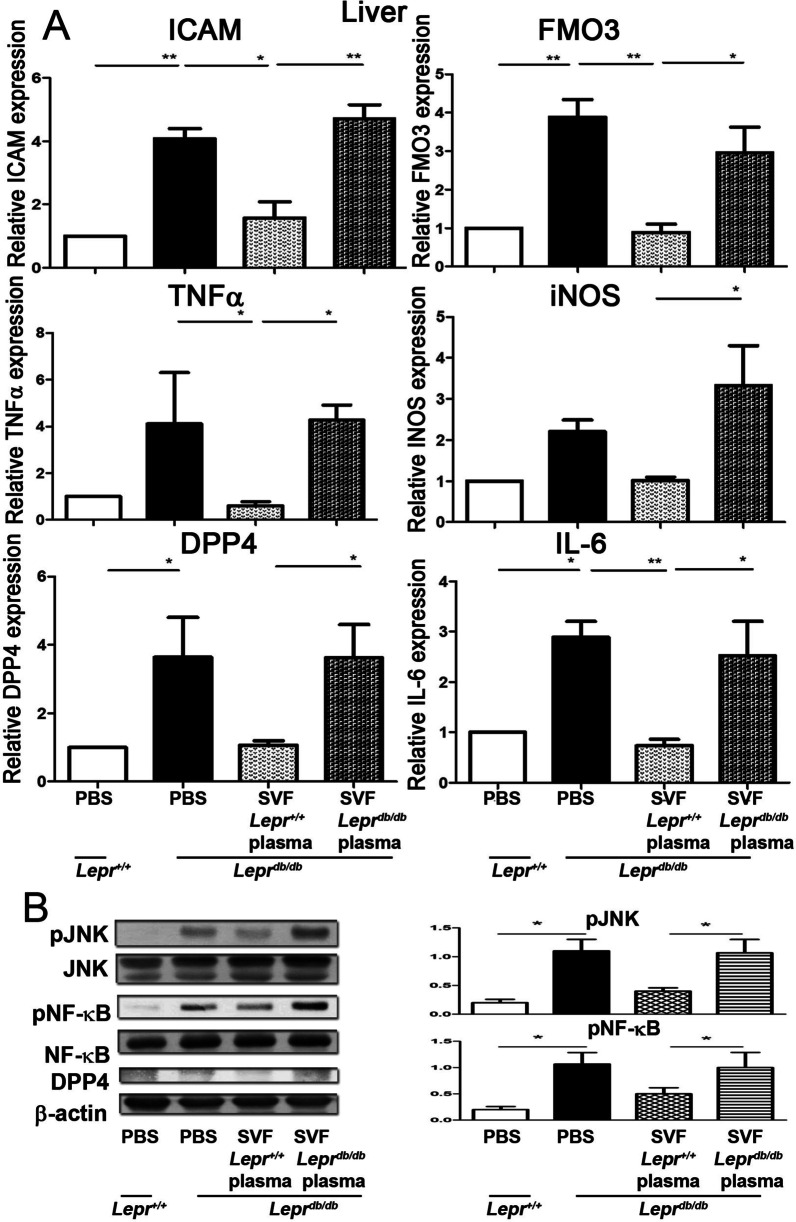
Nondiabetic plasma-treated SVFs decrease the expression of inflammatory meditators and suppress the activation of JNK and NFκB in the liver of *Lepr*^*db/db*^ mice. SVFs were harvested from the adipose tissue of *Lepr*^*db/db*^ mice and treated with plasma from *Lepr*^+*/*+^ or *Lepr*^*db/db*^ mice. *Lepr*^+*/*+^ or *Lepr*^*db/db*^ plasma-treated SVFs were injected into the inguinal WAT of *Lepr*^*db/db*^ mice. *Lepr*^+*/*+^ and *Lepr*.^*db/db*^ mice were also treated with PBS as controls. One week later, the liver was harvested and subjected to Q-PCR analysis for ICAM, FMO3, TNFα, iNOS, DPP4, and IL-6 mRNA expression (**A**), and western blot analysis for pJNK, JNK, pNFκB, NFκB, and DPP4 protein expression (**B**). *N* = 3–4/group. *p < 0.05; **p < 0.01

We examined whether nondiabetic plasma-treated SVFs modulate the expression of proinflammatory mediators in the liver of diabetic mice. *Lepr*^*db/db*^ SVFs treated with *Lepr*^+*/*+^ or *Lepr*^*db/db*^ plasma were injected into the inguinal WAT of *Lepr*^*db/db*^ mice, and the liver was harvested and subjected to Q-PCR analysis to determine expression of proinflammatory cytokines at day 7 post-injection. Our results showed a significant reduction of ICAM, TNFα, DPP4, FMO3, iNOS, and IL-6 in the liver of *Lepr*^*db/db*^ mice receiving the injection of *Lepr*^+*/*+^ plasma-treated SVFs compared to those receiving the injection of *Lepr*^*db/db*^ plasma-treated SVFs (Fig. 6A). We then examined whether nondiabetic plasma-treated SVFs alter JNK and NFκB activation in the liver of diabetic mice. Our results showed that the phosphorylation of JNK and NFκB was decreased in the liver of *Lepr*^*db/db*^ mice injected with *Lepr*^+*/*+^ plasma-treated SVFs compared to those injected with *Lepr*^*db/db*^ plasma-treated SVFs (Fig. 6B and Additional file 1: Fig. S1). Altogether, these results suggest that nondiabetic plasma-treated SVFs modulate inflammatory cytokine expression and suppress JNK and NFκB activation in the liver of diabetic mice.

### Nondiabetic plasma-treated SVFs decrease adipose tissue IL-33 and plasma CCL2 as well as adiponectin levels, and inhibit plasma DPP4 activity in diabetic mice

**Fig. 7:**
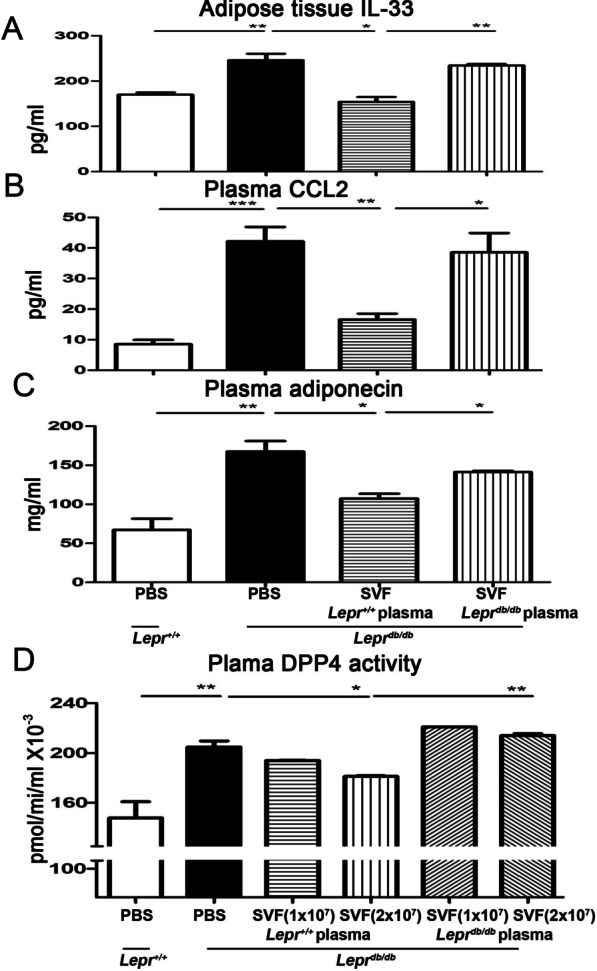
Nondiabetic plasma-treated SVFs decrease adipose tissue IL-33 expression, plasma CCL2 and adiponectin levels, and plasma DPP4 activity in diabetic mice. SVFs were harvested from the adipose tissue of *Lepr*^*db/db*^ mice and then treated with plasma from *Lepr*^+*/*+^ or *Lepr*^*db/db*^ mice. *Lepr*^+*/*+^ or *Lepr*^*db/db*^ plasma-treated SVFs were injected into the inguinal WAT of *Lepr*^*db/db*^ mice. *Lepr*^+*/*+^ and *Lepr*^*db/db*^ mice were also treated with PBS as controls. **A** One week later, the adipose tissue was harvested and then subjected to ELISA to detect IL-33 expression. The blood was harvested to determine plasma CCL2 **B** and adiponectin **C** levels. **D** *Lepr*^*db/db*^ mice were injected with increased numbers (1 × 10^7^ and 2 × 10^7^) of *Lepr*^+*/*+^ or *Lepr*.^*db/db*^ plasma-treated SVFs, and the blood was harvested to measure plasma DPP4 activity. N = 5/group. *p < 0.05; **p < 0.01; ***p < 0.001

To examine whether the injection of nondiabetic plasma-treated SVFs affect the level of IL-33 and CCL2 in adipose tissue and plasma in diabetic mice, respectively, SVFs treated with *Lepr*^+*/*+^ or *Lepr*^*db/db*^ plasma were injected into the inguinal WAT of *Lepr*^*db/db*^ mice, and the level of IL-33 in adipose tissue and CCL2 in plasma was measured. Our results showed that the injection of *Lepr*^+*/*+^ plasma-treated SVFs decreased the level of IL-33 in adipose tissue and CCL2 in plasma of *Lepr*^*db/db*^ mice compared to those with the injection of *Lepr*^*db/db*^ plasma-treated SVFs (Fig. 7A and B). Next, we examine the plasma adiponectin levels in different groups. Our results demonstrated that *Lepr*^*db/db*^ mice exhibited a higher plasma adiponectin level compared with *Lepr*^+*/*+^ mice (167.6 ± 13.33 vs. 67.27 ± 14.35 mg/ml) (Fig. 7C). The injection of *Lepr*^+*/*+^ plasma-treated SVFs into the adipose tissue of *Lepr*^*db/db*^ mice decreased the plasma adiponectin level compared to the injection of *Lepr*^*db/db*^ plasma-treated SVFs in *Lepr*^*db/db*^ mice (107.3 ± 6.09 vs. 141.4 ± 1.34 mg/ml) (Fig. 7C). We then investigated whether plasma DPP4 activity was affected by the injection of nondiabetic plasma- or diabetic plasma-treated SVFs. Under PBS treatment controls, we observed *Lepr*^*db/db*^ mice exhibited a higher plasma DPP4 activity compared with *Lepr*^+*/*+^ mice (204.8 ± 4.9 vs. 147.8 ± 13.06 pmol/min/ml × 10^−3^) (Fig. 7D). Notably, under SVF treatment conditions, the injection of increased number of *Lepr*^+*/*+^ plasma-treated SVFs into the adipose tissue of *Lepr*^*db/db*^ mice suppressed the plasma DPP4 activity compared to the injection of *Lepr*^*db/db*^ plasma-treated SVFs in *Lepr*^*db/db*^ mice (Fig. 7D). Altogether, these results demonstrate that the diabetic mice exhibit increased adipose tissue IL-33 and plasma CCL2 as well as adiponectin levels and enhanced plasma PDD4 activity, whereas the administration of nondiabetic plasma-treated SVFs is capable of suppressing adipose IL-33 and plasma CCL2 as well as adiponectin levels and inhibiting plasma DPP4 activity in diabetic mice compared to those with the administration of diabetic plasma-treated SVFs.

### Nondiabetic plasma-treated SVFs increase Akt activation and reverse glucose intolerance in ***Lepr***^***db/db***^ mice

**Fig. 8:**
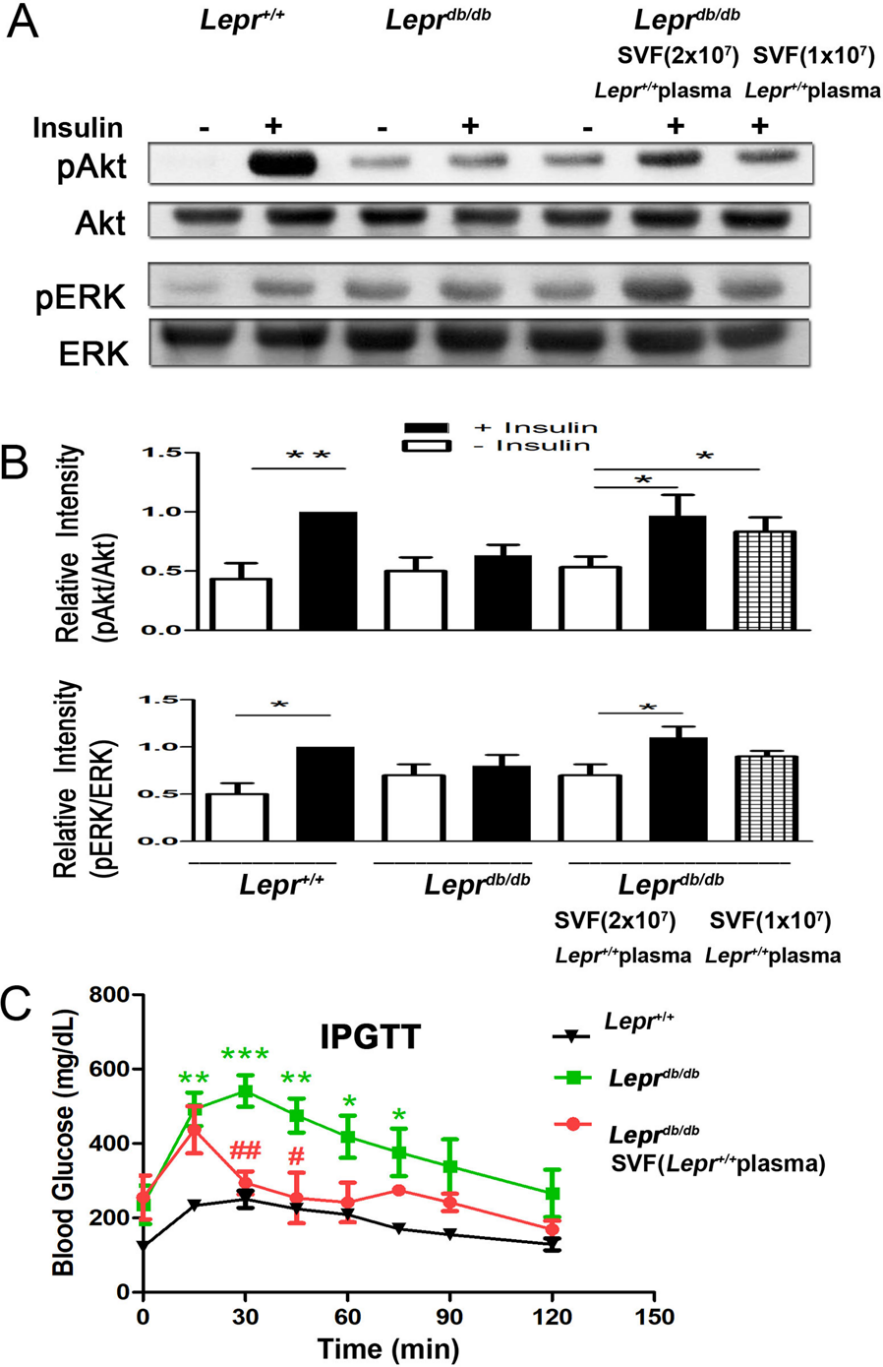
Nondiabetic plasma-treated SVFs increase Akt activation and attenuate glucose intolerance in *Lepr*^*db/db*^ mice. **A** *Lepr*^*db/db*^ SVFs were treated with plasma from *Lepr*^+*/*+^ mice. Increased numbers (1 × 10^7^ and 2 × 10^7^) of *Lepr*^+*/*+^ plasma-treated SVFs were injected into the inguinal WAT of *Lepr*^*db/db*^ mice. *Lepr*^+*/*+^ and *Lepr*^*db/db*^ mice treated with PBS were served as controls. One week after injection, mice were treated with vehicle or insulin 1.25 mIU/g body weight for 20 min. Mice were then killed and SVFs were harvested followed by western blot analysis for pAkt, Akt, pERK, and ERK expression. **B** The ratios of pAkt/Akt and pERK/ERK were calculated. **C** After 15 h fasting, *Lepr*^+*/*+^ mice treated with PBS, *Lepr*^*db/db*^ mice treated with PBS, and *Lepr*^*db/db*^ mice injected with *Lepr*^+*/*+^ plasma-treated SVFs were administered with glucose. The blood glucose level was measured before and every 15 min up to 2 h after glucose administration by using a glucose meter. *N* = 4/group. *p < 0.05; **p < 0.01; ***p < 0.001 compared to *Lepr*^+*/*+^ mice. ^#^*p* < 0.05; ^##^*p* < 0.01 compared to *Lepr*^*db/db*^ mice

To investigate whether the injection of nondiabetic plasma-treated SVFs attenuates insulin resistance in diabetic mice, *Lepr*^*db/db*^ mice were injected with *Lepr*^+*/*+^ plasma- or *Lepr*^*db/db*^ plasma-treated SVFs followed by insulin administration, and the Akt phosphorylation in the liver of *Lepr*^*db/db*^ mice was then measured. We observed a significant reduction of Akt phosphorylation in the liver of *Lepr*^*db/db*^ mice compared to that of *Lepr*^+*/*+^ mice following insulin administration (Fig. 8A and B), indicating a poor response to insulin treatment in *Lepr*^*db/db*^ mice. However, the injection of *Lepr*^+*/*+^ plasma-treated SVFs was able to induce Akt phosphorylation with a dose-dependent manner in the liver of *Lepr*^*db/db*^ mice following insulin administration (Fig. 8A and B, and Additional file 1: Fig. S2). In addition, the injection of *Lepr*^+*/*+^ plasma-treated SVFs enhanced ERK phosphorylation in the liver of *Lepr*^*db/db*^ mice following insulin administration (Fig. 8A and B, and Additional file 1: Fig. S2).

To further examine the effect of nondiabetic plasma-treated SVFs on diabetes-induced glucose intolerance, the glucose tolerance test was performed in *Lepr*^+*/*+^ and *Lepr*^*db/db*^ mice as well as in *Lepr*^*db/db*^ mice injected with nondiabetic plasma-treated SVFs. After fasting, *Lepr*^+*/*+^ mice showed an increased blood glucose level after glucose administration, and the blood glucose level returned to the normal level after 1 h of glucose administration. However, *Lepr*^*db/db*^ mice exhibited a significant higher blood glucose level throughout the first 75 min after glucose administration compared to *Lepr*^+*/*+^ mice (Fig. 8C). Notably, *Lepr*^*db/db*^ mice injected with nondiabetic plasma-treated SVFs displayed a significant reduction of blood glucose level compared to *Lepr*^*db/db*^ mice and had a similar level of blood glucose level compared to *Lepr*^+*/*+^ mice at 30 min after glucose administration (Fig. 8C). Altogether, these results suggest that the injection of nondiabetic plasma-treated SVFs enhances Akt activation and attenuates insulin resistance in *Lepr*^*db/db*^ mice.

## Discussion

Blocking ATM inflammation has been shown to improve the insulin sensitivity in obese mice [14]. The study by Ghorpade et al*.* demonstrated that 10% plasma from diet-induced obesity mice (DIO) induced a higher expression of monocyte chemoattractant protein 1 and IL-6 than 10% plasma from lean mice in SVFs of DIO mice [11]. Furthermore, the administration of ATMs from lean mice to obese recipients improved glucose tolerance and insulin sensitivity in obese mice through the secretion of miRNA-containing exosomes [15]. These results imply that the plasma from lean mice can repress the inflammatory cytokine expression in SVFs from obese mice, and the ATMs from lean mice attenuate insulin resistance in obese mice. Thus, in this study we examined the effects of adipose-derived cells on diabetes-induced adipose tissue inflammation, systemic inflammation, liver inflammation, and insulin resistance. We found that the injection of nondiabetic plasma-treated SVFs into the adipose tissue of diabetic mice significantly suppressed inflammatory M1 cytokines, including TNF-α, IL-6, IL-1β, IL-33, and DPP4, but enhanced anti-inflammatory M2 cytokine IL-10 expression in the adipose tissue of diabetic mice. In addition, the injection of nondiabetic plasma-treated SVFs not only repressed the expression of the diabetes-induced ICAM, FMO3, IL-6, IL-1β, iNOS, TNF-α, and DPP4 expression, but also suppressed the phosphorylation of JNK and NF-κB in the liver of diabetic mice. Furthermore, we found nondiabetic plasma-treated SVFs decreased adipose tissue IL-33 expression and plasma CCL22 level and suppressed plasma DPP4 activity in diabetic mice. Moreover, nondiabetic plasma-treated SVFs enhanced Akt activation in *Lepr*^*db/db*^ mice following insulin administration and attenuated glucose intolerance in *Lepr*^*db/db*^ mice. Collectively, we identify that the injection of nondiabetic plasma-conditioned diabetic SVFs is capable of mitigating diabetes-induced adipose inflammation, systemic inflammation, and insulin resistance and that may be mediated through inhibiting M1 but enhancing M2 cytokine expression in adipose tissue of diabetic mice.

Previous study demonstrates that IL-10 signaling is necessary for the normal suppression of gluconeogenic gene expression, and the physiological concentrations of IL-10 and insulin can suppress glucose production in primary hepatocytes [8]. Our data demonstrated that diabetes repressed IL-10 expression in the adipose tissue; however, nondiabetic plasma was able to induce IL-10 expression in SVFs isolated from diabetic mice. Moreover, we found that the injection of nondiabetic plasma-treated SVFs into the adipose tissue of diabetic mice increased IL-10 expression in the adipose tissue, decreased inflammatory cytokine expression in the liver, suppressed plasma DPP4 activity and CCL2 level, and improved insulin resistance. Altogether, these findings suggest that IL-10 signaling in adipose tissue may be required for normal suppression of insulin resistance, and nondiabetic plasma treatment of SVFs that stimulates IL-10 production could be used as a novel cell therapy strategy to decrease proinflammatory cytokine expressions in the adipose tissue and liver, reduce systemic CCL2 levels, and improve insulin resistance in diabetics.

An inflammatory state of WAT has been found in the adipose tissue of obese human and obesity animal models, and the adipose tissue was extensively infiltrated with immune cells, such as macrophages, dendritic cells, and lymphocytes [16]. Tregs play a critical role in modulating the adipose tissue inflammatory tone and maintaining insulin sensitivity [9]. Studies have showed that the population of Tregs in epididymal fat was markedly decreased in obese animals, and this reduction was closely associated with insulin resistance [10]. Tregs exert effects on the inhibition of effector T cell activation and proliferation, and the modulation of innate immune system activities. The depletion of Tregs in mice by diphtheria toxin has been shown to aggravate the adipose tissue inflammation and insulin resistance [17]. Moreover, the expansion of Tregs by the injection of IL-2 was able to decrease adipose tissue inflammation and improve insulin sensitivity through IL-10-mediated suppression of conventional T cell proliferation in mice [17]. Interestingly, although our data showed that the treatment of *Lepr*^+*/*+^ plasma did not alter the level of Foxp3 expression in *Lepr*^*db/db*^ SVFs compared to the treatment of *Lepr*^*db/db*^ plasma or PBS in vitro*,* the injection of *Lepr*^+*/*+^ plasma-treated SVFs significantly increased Foxp3 expression in the adipose tissue of diabetic mice compared to those treated with PBS in vivo. These results suggest that nondiabetic plasma does not directly induce Tregs in SVFs. Instead, nondiabetic plasma induces IL-10 expression in SVFs that may subsequently promote Treg expansion in the adipose tissue of diabetic mice. Thus, these findings corroborate that nondiabetic plasma-treated SVFs could be developed as a potential cell therapeutic approach for inducing Tregs in adipose tissue and improving insulin sensitivity in diabetes.

Previously, we demonstrated that SVFs from *Lepr*^*db/db*^ mice displayed increased JNK activation compared with those from *Lepr*^+*/*+^ mice. Injection of *Lepr*^+*/*+^ plasma mice into the adipose tissue of *Lepr*^*db/db*^ mice significantly decreased JNK activation in SVFs of *Lepr*^*db/db*^ mice compared with those receiving *Lepr*^*db/db*^ plasma [18]. These results suggest that diabetes induces JNK activation of adipose tissue and nondiabetic plasma diminishes diabetes-induced JNK activation. Previously, we demonstrated that diabetes increases intestinal iNOS expression, NO levels in the portal vein, and IL-1β and TNF-α expressions in liver Kupffer cells. The iNOS inhibition by L cells [19]. Here we demonstrated that the injection of nondiabetic plasma-treated SVFs into the adipose tissue of *Lepr*^*db/db*^ mice decreased proinflammatory cytokine expression, suppressed plasma DPP4 activity and CCL2 levels, and ameliorated insulin resistance. In addition, we found the frequency of CD11c^+^ CD11b^+^ M1 fraction increased in the adipose tissue of diabetic mice compared to that of nondiabetic mice, whereas the injection of nondiabetic plasma-treated SVFs into the adipose tissues of diabetic mice significantly decreased the frequency of CD11c^+^CD11b^+^ M1 cells. Altogether, these results suggest that nondiabetic plasma-treated SVFs injection into diabetic adipose tissues can be used to reverse the diabetes-induced systemic inflammation and insulin resistance through the decrease in the proinflammatory cytokine expression in the liver, and the injection of nondiabetic plasma-treated SVFs into adipose tissues can decrease M1 macrophages in the adipose tissues of diabetic mice.

Previous study demonstrate that DPP‐4 inhibitor treatment may prevent obesity through the activation of brown adipose tissue function [20]. It has been shown that DPP4 activity correlated with the onset and severity of obesity and diabetes [21], and plasma DPP4 activity was elevated in type 2 diabetes mellitus and obesity [22]. Similarly, we observed that diabetes-enhanced DPP4 expression in tissue and activity in plasma were closely related to diabetes-induced systemic inflammation and glucose intolerance. First, *Lepr*^*db/db*^ mice exhibited an increased DPP4 expression in the adipose tissue and liver compared with *Lepr*^+*/*+^ mice. Second, the in vitro treatment of Lepr^*db/db*^ SVFs with diabetic plasma significantly induced DPP4 expression compared with those treated with nondiabetic plasma. Third, the injection of nondiabetic plasma-treated SVFs decreased plasma DPP4 activity and repressed DPP4 expression in adipose tissue and liver of diabetic mice. Collectively, our findings reveal that the suppression of DPP4 expression in adipose tissue and DPP4 activity in plasma with nondiabetic plasma-treated SVFs may ultimately attenuate glucose intolerance and insulin resistance in diabetes.

## Conclusion

**Fig. 9:**
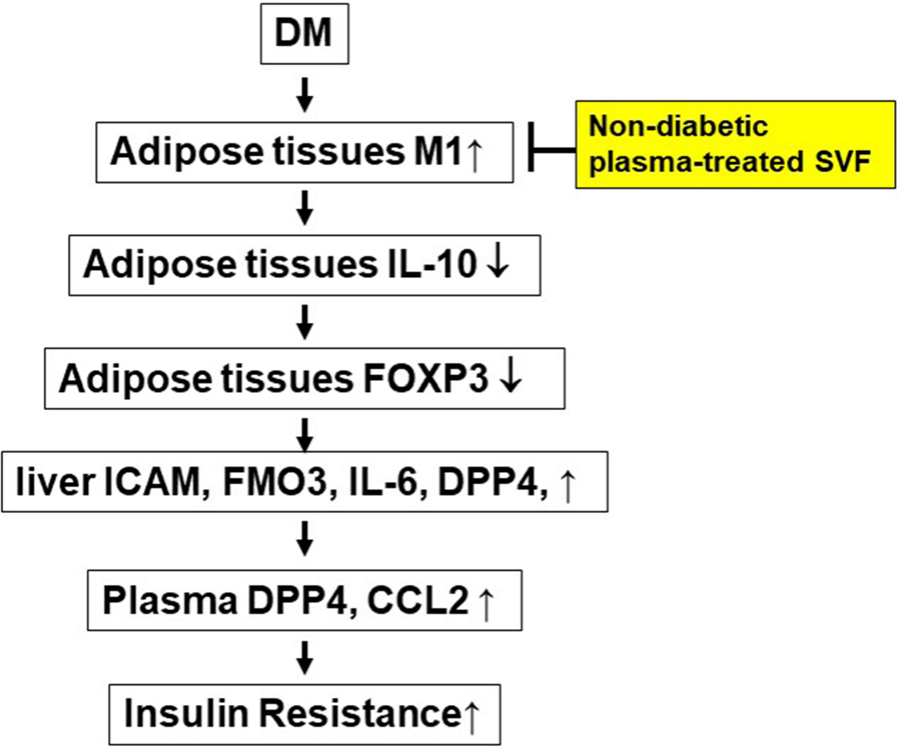
The model of nondiabetic plasma-treated SVFs modulating M1 expression and attenuating insulin resistance in diabetic mice. Diabetes induces M1 but decreases IL-10 cytokine expression in adipose tissue. Increased adipose tissue M1 expression enhances plasma DPP4 activity and induces liver inflammatory mediators, including ICAM, FMO3, IL-1β, iNOS, TNF-α, IL-6, and DPP4 expression. Subsequently, the increased inflammatory mediator expression and plasma DPP4 activity may promote insulin resistance and glucose intolerance in diabetic mice. Nondiabetic plasma-treated SVFs inhibit M1 expression, increase IL-10 and Foxp3 expression in the adipose tissue, decrease plasma DPP4 activity and CCL2 level, and suppress liver inflammatory mediator expression that, leading to attenuated insulin resistance and glucose intolerance in diabetic mice

In this work, we demonstrate the potential protective effects of adipose-derived cell treatment in modulating diabetes-induced DPP4 activity, insulin resistance, and systemic inflammation (Fig. 9). We found that diabetes-induced M1 cytokine expression in adipose tissue, promoted M1 cytokine expression and JNK and NF-κβ activation in liver, and enhanced plasma DPP4 activity that may subsequently promote insulin resistance and glucose intolerance in diabetic mice. However, nondiabetic plasma-treated SVFs were able to inhibit M1 expression and increase IL-10 expression in the adipose tissue of diabetic mice. The induction of IL-10 expression in the adipose tissue might promote Foxp3^+^ Treg expansion and suppress liver inflammatory cytokine expression and plasma DPP4 activity that may ultimately lead to mitigated insulin resistance and glucose intolerance in diabetic mice. Our results suggest that the cell therapeutic approach by using nondiabetic plasma-treated SVFs could be a promising strategy to attenuating DPP4 activity, systemic inflammation, and insulin resistance in diabetic patients.

## Supplementary Information

**Additional file 1. Supplemental Figure 1:** Non-diabetic plasma-treated SVFs modulate inflammatory cytokine expression, and suppress JNK and NFκB activation in the liver of diabetic mice. Uncropped Western blot images of p-JNK, JNK, p- NFκB, NFκB, DPP4, and β-actin of liver. Representative images and statistical analysis are presented in Figure 6B. **Supplemental Figure 2**: The injection of Lepr^+/+^ plasma-treated SVFs enhanced ERK phosphorylation in the liver of Lepr^db/db^ mice following insulin administration. Uncropped Western blot images of pAkt, Akt, pERK, and ERK of liver. Representative images and statistical analysis are presented in Figure 8A.

## Data Availability

All relevant data and material to reproduce the findings are available in the manuscript.

## Abbreviations

SVFs: Stromal vascular fractions
ATMs: Adipose tissue macrophages
NATMs: Non-ATMs
DPP4: Dipeptidyl peptidase 4
Tregs: Regulatory T cells
IPGTTs: Intraperitoneal glucose tolerance tests (IPGTTs) (Tregs)
FMO: Flavin mono-oxygenase
CCL2: Chemokine (CC-motif) ligand 2
ICAM: Intercellular adhesion molecule
TNF-α: Tumor-necrosis factor (TNF-α)
iNOS: Inducible nitric oxide synthase
JNK: C-Jun N-terminal kinases
IL-1β: Interleukin 1 beta
