# Supplementary material for: Adipose-derived stromal cells reverse insulin resistance through inhibition of M1 expression in a type 2 diabetes mellitus mouse model
Source: Stem Cell Res Ther. 2022 Jul 26;13:357. doi: 10.1186/s13287-022-03046-0 (PMC9327360; doi:10.1186/s13287-022-03046-0)

**Adipose-derived stromal cells reverse insulin resistance through inhibition of M1 expression in a type 2 diabetes mellitus mouse model**

Lee-Wei Chen^1, 2, 3*^, Pei-Hsuan Chen^1^, Chia-Hua Tang^1^, Jui-Hung Yen^4*^

**Supplemental Figures**

**Supplemental Figure 1:** Non-diabetic plasma-treated SVFs modulate inflammatory cytokine expression, and suppress JNK and NFκB activation in the liver of diabetic mice. Uncropped Western blot images of p-JNK, JNK, p- NFκB, NFκB, DPP4, and β-actin of liver. Representative images and statistical analysis are presented in Figure 6B.

p-JNK


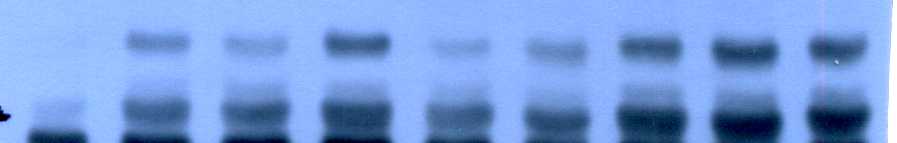


JNK


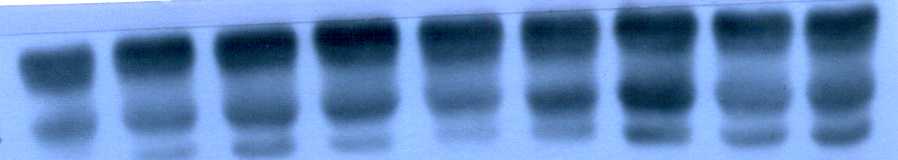


p-NFkB


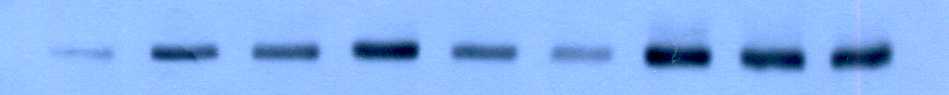


NF-kB


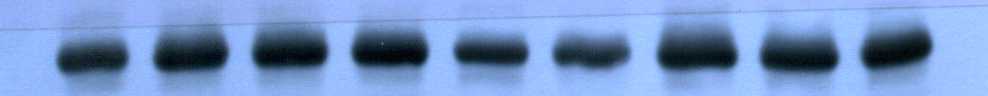


DPP4


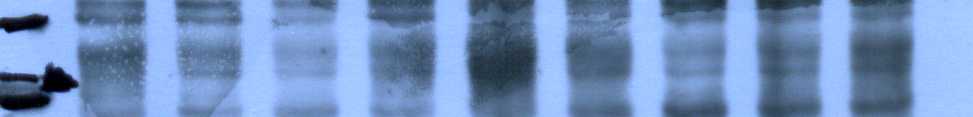


B-actin


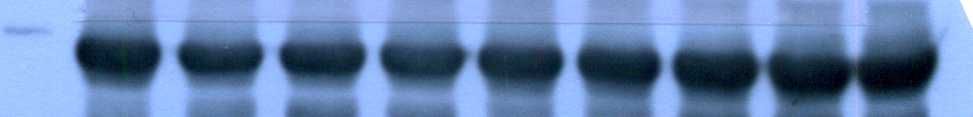


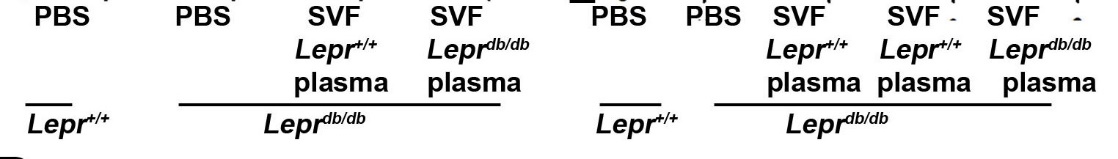


**Supplemental Figure 2:** The injection of *Lepr^+/+^* plasma-treated SVFs enhanced ERK phosphorylation in the liver of *Lepr^db/db^* mice following insulin administration. Uncropped Western blot images of pAkt, Akt, pERK, and ERK of liver. Representative images and statistical analysis are presented in Figure 8A.

pAkt


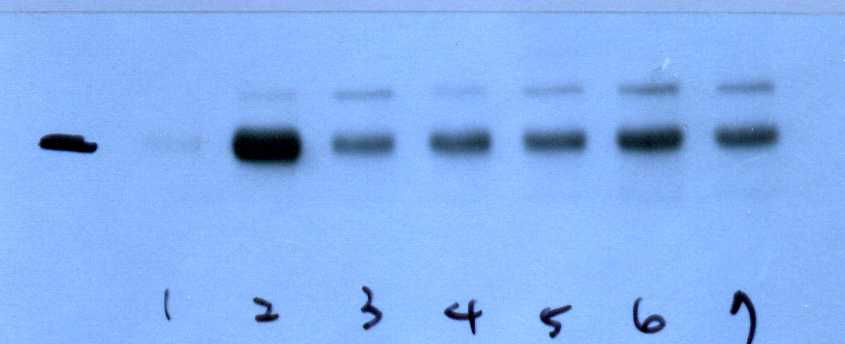


pAkt


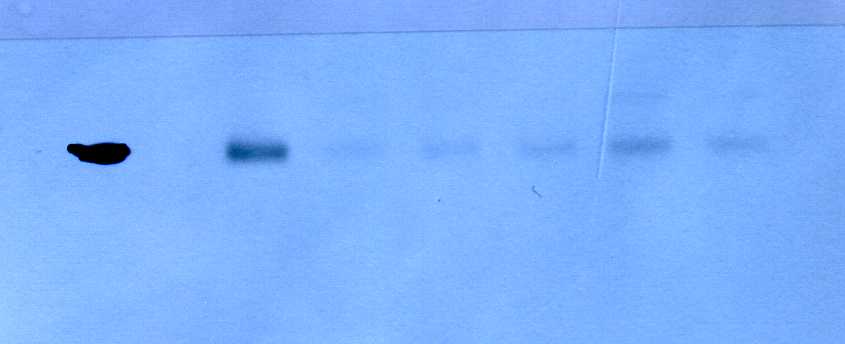


Akt


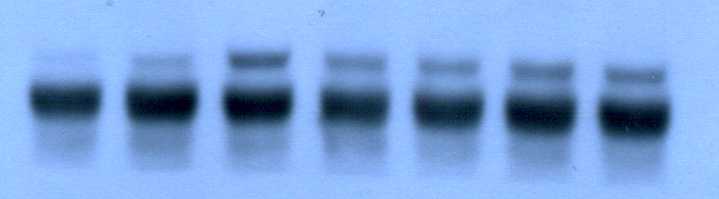


pERK


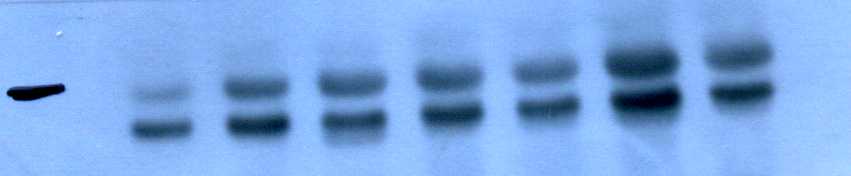


ERK


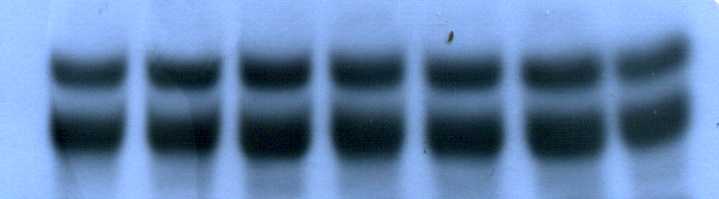

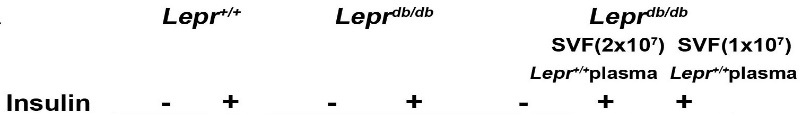


pAkt


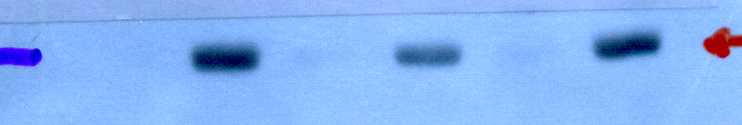


Akt


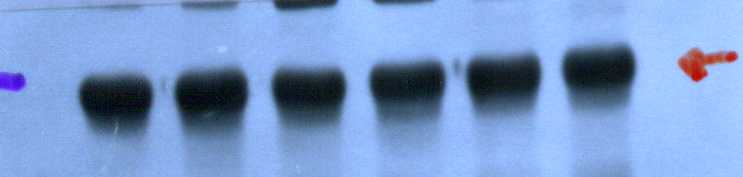


ERK


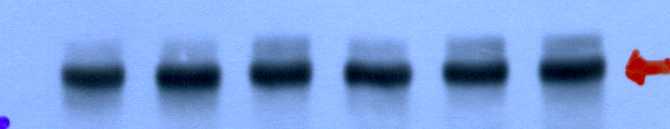


pERK


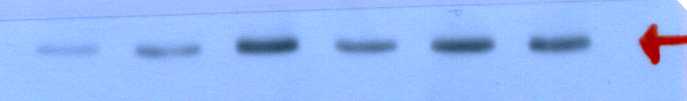


b-actin


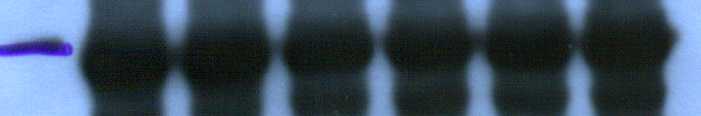


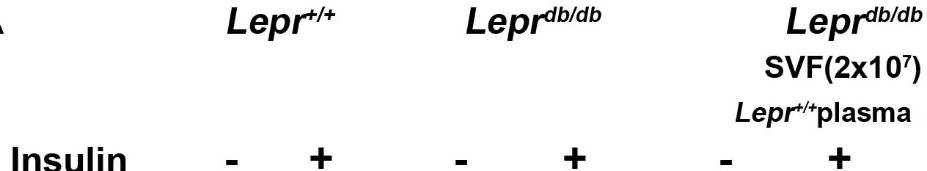

Supplement: Supplementary file 1 — Additional file 1. Supplemental Figure 1: Non-diabetic plasma-treated SVFs modulate inflammatory cytokine expression, and suppress JNK and NFκB activation in the liver of diabetic mice. Uncropped Western blot images of p-JNK, JNK, p- NFκB, NFκB, DPP4, and β-actin of liver. Representative images and statistical analysis are presented in Figure 6B. Supplemental Figure 2: The injection of Lepr+/+ plasma-treated SVFs enhanced ERK phosphorylation in the liver of Leprdb/db mice following insulin administration. Uncropped Western blot images of pAkt, Akt, pERK, and ERK of liver. Representative images and statistical analysis are presented in Figure 8A. [file 13287_2022_3046_MOESM1_ESM.docx]
